# Educators as agents of breadth-biased learning: using social reconstructionism as rationale for embracing media multitasking and enhancing teaching practices in higher education

**DOI:** 10.3389/fpsyg.2024.1356232

**Published:** 2024-04-03

**Authors:** Seada A. Kassie

**Affiliations:** Department of Psychology, School of Science and Technology, Middlesex University Dubai, Dubai, United Arab Emirates

**Keywords:** media multitasking, cognitive flexibility, breadth-biased learning, mindfulness practices, higher education, learning and teaching, pedagogical approaches

## Abstract

This perspective article contends that media multitasking has significant implications on cognitive control processes, particularly in how information is processed and utilized. Contrary to viewing media multitasking as inherently negative, the article argues that it contributes to the evolving nature of cognitive processing, without necessarily improving or degrading it. The discussion draws on theoretical frameworks from contemporary cognitive neuroscience to contextualize these arguments. The article provides a nuanced perspective on media multitasking, acknowledging its enduring presence and exploring its influence on cognitive processes, while also proposing strategies for educators to navigate its implications in educational settings.

## Introduction

The philosophy of social reconstructionism challenges the status quo of education and invites educators to take agency of societal change. This prestigious agency is granted to educators based on the notion that every educated member of society is a product of schools, and as such, should be equipped with the right skills and resources to bring about lasting solutions to social problems. In this perspective piece, I use the philosophy of social reconstructionism to critically evaluate the popular notion that, as media multitasking becomes part of everyday life, infiltrating both living, learning, and working spaces, it is bringing with it negative consequences such as superficial mode of acquiring information and knowledge, as well as reducing accuracy and performance of cognitive tasks (Schuur et al., 2015). The article aims to provide a rationale for embracing the here-to-stay nature of media multitasking and endorsing the argument that it in fact influences the evolving nature of cognitive control processes, in that it alters the way information is received and utilized, without necessarily making it better or worse. Theoretical frameworks from recent and current practices in cognitive neuroscience and computational modeling are discussed to provide contextual basis for the key arguments in the article. Primary focus is given to a critical discussion on cognitive processes involved in media multitasking, their direct implications on learning and education, and practical solutions for educators to enhance teaching practices in the new media age.

## Social reconstructionism

Social reconstructionism is a philosophy of education founded by Brameld (1977) who advocated for empowering teachers with the tools and resources they need to bring about enduring solutions to fundamental problems at the school level in particular and the societal level in general (Liston and Zeichner, 1991). The theory aims to understand the historical foundations of social issues and encourages educators to take the lead in reforming the ways in which schools provide education and produce effective generations of leaders. In so doing, social reconstructionism empowers educators to oppose perpetuating the status quo and instead become agents of social change (Parks, 2006; Ong'ondo, 2017). At the core of social reconstructionism is the belief that the classroom is where educators and students identify social priorities and continuously work toward finding implementable solutions for them. Identifying current day media multitasking as a societal priority and one that is particularly impacting students in higher education, this article encourages institutions to adapt to the way the brain processes information and find congruency between the way educators deliver their teaching and the manner students receive it.

## Chronic media-multitasking

Chronic media multitasking is causing structural changes in the brain (May and Elder, 2018). Given the age we live in, there is a growing body of research on media multitasking and its effect on the way the brain processes information (Coulacoglou and Saklofske, 2017; Firth et al., 2019; Korte, 2020). As a relatively new area of research, media multitasking is motivating researchers to explore the possible novel ways that the cognitive system is encoding, processing, and utilizing information. Studies have found varying levels of media use across different age groups, where young adults are found to be the heaviest media multitaskers (Carrier et al., 2009; Courage et al., 2015; Uncapher et al., 2017). They are also the focus of studies due to developmental research showing the brain's malleability and susceptibility to environmental stimuli during this key developmental stage (Arnett, 2000). It is argued that by virtue of neuroplasticity, emerging adults are at an advantage for increased environmental adaptability; and accordingly, extensive and chronic media use as experienced by this age group may have an association with developmental neuroplasticity, the brain's capacity to rewire and form new neural pathways (Firth et al., 2019; Korte, 2020).

## Breadth-biased information processing

There is growing interest in how chronic media multitasking might be altering the way the brain processes information, from a linear and in-depth cognitive processing to a more breadth-biased one (Foerde et al., 2006; Poldrack and Foerde, 2007; Lin, 2009; Ophir et al., 2009; Uncapher et al., 2017). Contrary to the former, breadth-biased processing is characterized by engaging the brain in multiple activities and paying attention to several sources of information simultaneously (Lin and Parsons, 2018). Studies have found that heavy media multitaskers process information in a breadth-biased manner (Karpinski et al., 2013; Poplawska et al., 2021), which is quite distinct from the linear and in-depth style. This shift is attributed to chronic media consumption, which is increasingly becoming part of current day learning practices. Breadth-biased cognitive control involves information processing that is characterized by superficial and scattered attention toward several sources of information simultaneously, without focusing deeply on one single task. Ophir et al. (2009) argued that heavy media multitaskers are inclined to pay attention to a wider scope of information instead of focusing on a particular single piece. This may not necessarily mean inefficient use of cognitive resources, but rather a shift in cognitive control processes as chronic media consumption becomes part of everyday life.

Furthermore, in neuro-ergonomics, functional MRI studies have shown changes that occur in active parts of the brain during multitasking. Studies by Poldrack and Foerde (2007) and Foerde et al. (2013) found that the medial temporal lobe region of the brain, associated with declarative memory, is activated when selective and focused single task learning takes place; whereas the striatum, which is associated with procedural memory is activated when breadth-biased dual task learning takes place. In addition, the same studies showed increased activity around the dorsolateral pre-frontal cortex region when dual tasks were performed, which is a functional structure of the brain associated with media multitasking involving working memory capacity, cognitive flexibility, and abstract reasoning. The authors explain how in the presence of a distraction - in this particular case a secondary task - the results indicate that accuracy of performance is not affected; however, a shift occurs in the way the brain acquires learning, corresponding with the argument that multitasking might be altering the way the brain processes information.

Of the types of cognitive processes implicated in learning and the education environment, attention, working memory, cognitive control, and executive functioning are the notable ones (Clark and Harrelson, 2002). Studies have shown that these same processes are influenced by media multitasking, and alterations in brain regions such as the prefrontal cortex, the anterior cingulate cortex, and the dorsolateral prefrontal cortex have been observed using neuroimaging techniques (Loh and Kanai, 2014; Verghese et al., 2016; Cao et al., 2022; Luo et al., 2022). While these studies indicate that heavy media multitaskers engaged in specific learning-related cognitive activities showed significantly worse executive function than their counterparts, they had greater prefrontal activation and did not perform significantly worse on cognitive tasks (Luo et al., 2022). Moreover, experimental studies focused on training in multitasking activities indicate significant training-induced performance improvements in the left prefrontal cortex and the dorsolateral prefrontal cortex, particularly in sensitivity to multitasking demands and heightened neural activity when performing single tasks regardless of variations in input/output modalities (Verghese et al., 2016). These findings have direct educational implications on higher learning where educators could embrace novel ways of teaching practices that extend beyond the traditional mode of teaching delivery.

## Mindfulness

Mindfulness experts argue that chronic media use is a defining characteristic of the modern digital world, hence inescapable, and scientific studies should instead focus on possible ways of enabling the cognitive system to cope with media use in an efficient and conducive manner through the practice of mindfulness (Brown and Ryan, 2003; Case and King, 2003; Carrier et al., 2009; Chiesa et al., 2011; Ie et al., 2013). Whilst trait mindfulness is a dispositional state of being, studies show that it can be enhanced and strengthened through various instructional intervention techniques. This, they argue, could foster cognitive flexibility and creative thinking, which are critical to learning in higher education (Langer and Moldoveanu, 2000; Sternberg, 2000; Hart et al., 2013; Rogaten and Moneta, 2016; Kercook et al., 2017). Moreover, the use of technology to deliver and access education has become increasingly mainstream and students are increasingly engaged in media multitasking to navigate through their education. Studies show that young adults who are also heavy media multitaskers could benefit from short-term mindfulness interventions whereby their attentional abilities are increased (Gorman and Green, 2016). Experimental research findings (Ie et al., 2013; Seddon et al., 2021) measuring cognitive performance on media multitasking activities found heavy media multitaskers who also had mindfulness as a dispositional trait performed significantly better. Arguably, this could be due to frequent media multitasking, resulting in significant improvement in cognitive flexibility.

## Cognitive flexibility

Cognitive flexibility is defined as the brain's ability to remain flexible in the face of a constantly changing environment (Cools, 2015; Feng et al., 2020). Advocates of mindfulness suggest that through frequent engagement in mindfulness practices, it is possible to alter the structures of the brain responsible for executive functioning and consequently develop more efficiency in cognitive capacity while engaging in multitasking activities (Moore and Malinowski, 2009; Ie et al., 2013; Seddon et al., 2021). Researchers are increasingly recommending ways of fostering mindfulness practices to increase cognitive flexibility and enhance multitasking abilities, both of which could benefit students' learning and academic performance (Coulacoglou and Saklofske, 2017).

In recent years, studies have focused on investigating the relationship between trait mindfulness and its effects on attention, working memory, and other cognitive functions (Corti and Gelati, 2020; Nassif et al., 2023). Functional Magnetic Resonance Imaging (fMRI) studies on emerging adults show dramatic maturity of the dorsolateral prefrontal cortex region of the brain, associated with cognitive control (Jaeger et al., 2012; Tymofiyeva and Gaschler, 2020). There is also greater functional connectivity of this region with the right hippocampus, posterior and anterior cingulate, and two areas of the prefrontal cortex, suggesting maturity in working memory capacity and cognitive control of emerging adults. What such findings reveal is not only the structural changes this particular age group undergoes developmentally, but also possibly the functional ways in which thinking, learning, and general acquisition of information are being altered in the face of external environmental stimuli, such as chronic media usage.

## General discussion and recommendations for educators

In the context of higher education, the discussion around media multitasking is essential due to its pervasive influence on students' learning approaches and the need for enhanced and evidence-based pedagogical practices. Firstly, educators could benefit from learning about the cognitive and neural mechanisms underlying media multitasking and its effects on the brain. Understanding the concepts discussed in this article, including breadth-biased processing, mindfulness, and cognitive flexibility is crucial for devising effective pedagogical strategies that take into account the effects of media multitasking on learning and cognition. Secondly, aligning pedagogical practices to the neural expectations brought on by media multitasking can play a vital role in enhancing cognitive flexibility and other desirable cognitive processes that aid learning. Educators can implement practical pedagogical strategies that promote engagement and enhance cognitive flexibility, including the following.

1. Provide mindfulness resources: Offer resources such as guided meditation recordings and mindfulness applications that students can access independently. Longitudinal studies have shown the positive and transformative impact of accessing mindfulness resources in higher education (Oberski et al., 2015; Barker et al., 2021; Ergas and Hadar, 2023).
2. Metacognitive strategies: Teach metacognitive strategies such as self-reflection, goal-setting, and strategic planning to help students develop awareness of their own thinking processes and learning strategies. Doing so has been found to enhance the ability to organize and integrate a large body of information, such as what is found when media multitasking, which in turn fosters creativity and cognitive flexibility (Meltzer, 2013).
3. Foster reflective practices and self-regulated learning: Encourage students to reflect on their learning experiences and identify instances in their learning journey where mindfulness practices could be beneficial. Teaching self-regulation strategies such as setting intentions and monitoring attentional focus has been found to augment cognitive flexibility and cognitive control among students (Marcovitch et al., 2008; Orakçi, 2021; Daggöl, 2023).
4. Promote problem-based learning: Implement problem-based learning (PBL) approaches where students work collaboratively to solve complex, real-world problems. PBL encourages students to explore multiple perspectives, consider alternative solutions, and adapt their thinking based on new information, fostering lateral and critical thinking skills (Tursynkulova et al., 2023).
5. Encourage Socratic questioning: Use Socratic questioning techniques to encourage students to critically analyze and evaluate ideas, arguments, and assumptions. By posing thought-provoking questions that challenge students' assumptions and encourage deeper exploration of topics, educators can promote meaningful learning and intellectual growth (Tursynkulova et al., 2023).
6. Provide experiential learning opportunities: This would help engage students in authentic, real-world experiences. Experiential learning encourages students to apply theoretical knowledge in practical contexts, adapt to novel situations, and learn from failure and iteration, which are at the heart of cognitive flexibility (Durak, 2023).
7. Assign problem-based media tasks: Present students with problem-based tasks that require them to navigate and evaluate information from multiple media sources to solve real-world challenges. For instance, students could analyze conflicting news reports on a current event, assess the credibility of online sources, and synthesize their findings to form evidence-based conclusions. This cultivates cognitive flexibility by challenging students to critically evaluate and integrate information from diverse media sources, mirroring the cognitive demands of media multitasking (DeHaan, 2009; Fjaellingsdal et al., 2021; Green and Rathgeb-Schnierer, 2023).

## Conclusion

In conclusion, media multitasking may have an effect on the evolving nature of cognitive control processes. Congruent with the theory of cognitive flexibility within the mindfulness literature, such concepts lend credence to the notion that media multitasking may be promoting a breadth-biased style of information processing. The philosophy of social reconstructionism grants educators the agency for social change, and institutions in higher education carry the responsibility to ensure that teaching methodologies are aligned with the most efficient ways that students can receive and process information. Experimental research findings (Corti and Gelati, 2020; Nassif et al., 2023) show that mindfulness practices and other teachable strategies can be developed without necessarily having prior experiences. The overarching argument here is that fostering mindfulness, and by extension, cognitive flexibility, will enhance breadth-biased learning, which will have a knock-on effect on students overall learning experiences and academic achievements in today's media-saturated world.

## Data availability statement

The original contributions presented in the study are included in the article/supplementary material, further inquiries can be directed to the corresponding author.

## Author contributions

SK: Conceptualization, Methodology, Project administration, Resources, Visualization, Writing – original draft, Writing – review & editing.

## References

[B1] Arnett J. J. (2000). Emerging adulthood: a theory of development from the late teens through the twenties. Am. Psychol. 55, 469–480. 10.1037/0003-066X.55.5.469 10842426

[B2] Barker R. K. Tuominen L. P. Larson M. R. Lee-Nichols M. E. Eslinger G. Patterson K. L.. (2021). Enhancing mindfulness and well-being in higher education. Int. J Commun. Well-Being 4, 625–646. 10.1007/s42413-021-00118-6 PMC8130210 34723120

[B3] Brameld T. (1977). Reconstructionism as radical philosophy of education: a reappraisal. Educ. Forum 42, 67–76. 10.1080/00131727709338153

[B4] Brown K. W. Ryan R. M. (2003). The benefits of being present: mindfulness and its role in psychological well-being. J. Pers. Soc. Psychol. 84, 822–848. 10.1037/0022-3514.84.4.822 12703651

[B5] Cao N. Pi Y. Qiu F. Wang Y. Xia X. Liu Y.. (2022). Plasticity changes in dorsolateral prefrontal cortex associated with procedural sequence learning are hemisphere-specific. NeuroImage 259:119406. 10.1016/j.neuroimage.2022.119406 35752417

[B6] Carrier L. M. Cheever N. A. Rosen L. D. Benitez S. Chang J. (2009). Multitasking across generations: multitasking choices and difficulty ratings in three generations of Americans. Comput. Hum. Behav. 25, 483–489. 10.1016/j.chb.2008.10.012

[B7] Case C. J. King D. I. (2003). Are undergraduates using the internet productively? Issues Inf. Syst. 5, 45–51.

[B8] Chiesa A. Calati R. Serretti A. (2011). Does mindfulness training improve cognitive abilities? A Systematic review of neuropsychological findings. Clin. Psychol. Rev. 31, 449–464. 10.1016/j.cpr.2010.11.003 21183265

[B9] Clark R. Harrelson G. L. (2002). Designing instruction that supports cognitive learning processes. J. Athletic Train. 37, S152–S159. 12937537 PMC164417

[B10] Cools R. (2015). Neuropsychopharmacology of cognitive flexibility. Brain Mapping: An Encyclopedic Reference 3, 349–353. 10.1016/B978-0-12-397025-1.00253-0

[B11] Corti L. Gelati C. (2020). Mindfulness and coaching to improve learning abilities in university students: a pilot study. Int. J. Environ. Res. Pub. Health 17:1935. 10.3390/ijerph17061935 32188028 PMC7142624

[B12] Coulacoglou C. Saklofske D. H. (2017). Executive Function, Theory of Mind, and Adaptive Behavior. Psychometrics and Psychological Assessment. Ontario: Academic Press.

[B13] Courage M. L. Bakhtiar A. Fitzpatrick C. Kenny S. Brandeau K. (2015). Growing up multitasking: the costs and benefits for cognitive development. Dev. Rev. 35, 5–41. 10.1016/j.dr.2014.12.002

[B14] Daggöl G. D. (2023). Online self-regulated learning and cognitive flexibility through the eyes of English-major students. Acta Educ. Generalis 13, 107–132. 10.2478/atd-2023-0006

[B15] DeHaan R. L. (2009). Teaching creativity and inventive problem solving in science. CBE—Life Sci. Educ. 8, 172–181. 10.1187/cbe.08-12-0081 19723812 PMC2736021

[B16] Durak H. Y. (2023). Examining various variables related to authentic learning self-efficacy of university students in educational online social networks: creative self-efficacy, rational experiential thinking, and cognitive flexibility. Curr. Psychol. 42, 22093–22102. 10.1007/s12144-022-03211-x 35729901 PMC9188916

[B17] Ergas O. Hadar L. L. (2023). Does mindfulness belong in higher education? An eight year research of students' experiences. Pedagogy Culture Soc. 31, 359–377. 10.1080/14681366.2021.1906307

[B18] Feng X. Perceval G. J. Feng W. Feng C. (2020). High cognitive flexibility learners perform better in probabilistic rule learning. Front. Psychol. 11:415. 10.3389/fpsyg.2020.00415 32231624 PMC7083211

[B19] Firth J. Torous J. Stubbs B. Firth J. A. Steiner G. Z. Smith L.. (2019). The “online brain”: how the internet may be changing our changing. World Psychiatry 18, 119–129. 10.1002/wps.20617 31059635 PMC6502424

[B20] Fjaellingsdal T. G. Vesper C. Fusaroli R. Tylén K. (2021). Diversity Promotes Abstraction and Cognitive Flexibility in Collective Problem Solving. Available online at: osf.io/umhf6

[B21] Foerde K. Knowlton B. J. Poldrack R. A. (2006). Modulation of competing memory systems by distraction. Proc. Nat. Acad. Sci. U. S. A. 103, 11778–11783. 10.1073/pnas.0602659103 16868087 PMC1544246

[B22] Foerde K. Race E. Verfaellie M. Shohamy D. (2013). A role for the medial temporal lobe in feedback-driven learning: evidence from amnesia. J. Neurosci. 33, 5698–5704. 10.1523/JNEUROSCI.5217-12.2013 23536083 PMC3865542

[B23] Gorman T. E. Green C. S. (2016). Short-term mindfulness intervention reduces the negative attentional effects associated with heavy media multitasking. Sci. Rep. 6:24542. 10.1038/srep24542 27086504 PMC4834474

[B24] Green M. Rathgeb-Schnierer E. (2023). Summing up: cognitive flexibility and mental arithmetic. J. Mathematics Educ. 13, 1–17. 10.26711/0075771527900

[B25] Hart R. Ivtzan I. Hart D. (2013). Mind the gap in mindfulness research: a comparative account of the leading schools of thought. Rev. Gen. Psychol. 17, 453–466. 10.1037/a0035212

[B26] Ie A. Haller C. S. Langer E. J. Courvoisier D. (2013). Mindful multitasking: the relationship between mindful flexibility and media multitasking. Comput. Hum. Behav. 28, 1526–1532. 10.1016/j.chb.2012.03.022

[B27] Jaeger A. Selmeczy D. O'Connor A. R. Diaz M. Dobbins I. G. (2012). Prefrontal cortex contributions to controlled memory judgment: fMRI evidence from adolescents and young adults. Neuropsychologia 50, 3745–3756. 10.1016/j.neuropsychologia.2012.10.022 23127796 PMC3518586

[B28] Karpinski A. C. Kirschner P. A. Ozer I. Mellot J. A. Ochwo P. (2013). An exploration of social networking site use, multitasking, and academic performance among United States and European university students. Comput. Hum. Behav. 29, 1182–1192. 10.1016/j.chb.2012.10.011

[B29] Kercook S. Lineweaver T. T. Frank C. C. Fromm E. D. (2017). Cognitive flexibility and its relationship to academic achievement and career choice of college students with and without attention deficit hyperactivity disorder. J. Postsecondary Educ. Disability 30, 327–342.

[B30] Korte M. (2020). The impact of the digital revolution on human brain and behavior: where do we stand? Dialogues Clin. Neurosci. 22, 101–111. 10.31887/DCNS.2020.22.2/mkorte 32699510 PMC7366944

[B31] Langer E. J. Moldoveanu M. (2000). The construct of mindfulness. J. Soc. Issues 56, 1–9. 10.1111/0022-4537.00148

[B32] Lin L. (2009). Breadth-biased versus focused cognitive control in media multitasking behaviors. Proc. Nat. Acad. Sci. U. S. A. 106, 15521–15522. 10.1073/pnas.0908642106 19805207 PMC2747151

[B33] Lin L. Parsons T. D. (2018). Ecologically valid assessments of attention and learning engagement in media multitaskers. TechTrends 62, 518–524. 10.1007/s11528-018-0311-8

[B34] Liston D. Zeichner K. (1991). Teacher Education and the Social Conditions of Schooling. New York, NY: Routledge.

[B35] Loh K. K. Kanai R. (2014). Higher media multi-tasking activity is associated with smaller gray-matter density in the anterior cingulate cortex. PloS ONE 9:e106698. 10.1371/journal.pone.0106698 25250778 PMC4174517

[B36] Luo B. Li J. Liu J. Li F. Gu M. Xiao H.. (2022). Frequency-dependent plasticity in the temporal association cortex originates from the primary auditory cortex, and is modified by the secondary auditory cortex and the medial geniculate body. J. Neurosci. 42, 5254–5267. 10.1523/JNEUROSCI.1481-21.2022 35613891 PMC9236291

[B37] Marcovitch S. Jacques S. Boseovski J. J. Zelazo P. D. (2008). Self-reflection and the cognitive control of behavior: Implications for learning. Mind Brain Educ. 2, 136–141. 10.1111/j.1751-228X.2008.00044.x

[B38] May K. E. Elder A. D. (2018). Efficient, helpful, or distracting? A literature review of media multitasking in relation to academic performance. Int. J. Educ. Technol. Higher Educ. 15, 1–15. 10.1186/s41239-018-0096-z

[B39] Meltzer L. (2013). Teaching Executive Functioning Processes: Promoting Metacognition, Strategy Use, and Effort. Handbook of Executive Functioning. New York, NY: Springer New York.

[B40] Moore A. Malinowski P. (2009). Meditation, mindfulness and cognitive flexibility. Consciousness Cognit. 18, 176–186. 10.1016/j.concog.2008.12.008 19181542

[B41] Nassif T. H. Adrian A. L. Gutierrez I. A. Dixon A. C. Rogers S. L. Jha A. Adler A. B. (2023). Optimizing performance and mental skills with mindfulness-based attention training: two field studies with operational units. Military Med. 188, e761–e770. 10.1093/milmed/usab380 34557922

[B42] Oberski I. Murray S. Goldblatt J. DePlacido C. (2015). Contemplation and mindfulness in higher education. Global Innov. Teaching Learn. Higher Educ. Transg. Boundaries 12, 317–340. 10.1007/978-3-319-10482-9_19

[B43] Ong'ondo C. O. (2017). Teacher education as an agent of social change: analysis of the Kenyan case. J. Educ. Soc. Policy 4, 147–155.

[B44] Ophir E. Nass C. Wagner A. D. (2009). Cognitive control in media multitaskers. Proc. Nat. Acad. Sci. U. S. A. 106, 15583–15587. 10.1073/pnas.0903620106 PMC2747164 19706386

[B45] Orakçi S. (2021). Exploring the relationships between cognitive flexibility, learner autonomy, and reflective thinking. Thinking Skills Creativity 41:100838. 10.1016/j.tsc.2021.100838

[B46] Parks M. W. (2006). I am from a very small town: social reconstructionism and multicultural education. Multic. Persp. 8, 46–50. 10.1207/s15327892mcp0802_8

[B47] Poldrack R. A. Foerde K. (2007). Category learning and the memory systems debate. Neurosci. Biobehav. Rev. 32, 197–205. 10.1016/j.neubiorev.2007.07.007 17869339

[B48] Poplawska A. Szumowska E. Kus J. (2021). Why do we need media multitasking? A self-regulatory perspective. Front. Psychol. 12:624649. 10.3389/fpsyg.2021.624649 33643153 PMC7905209

[B49] Rogaten J. Moneta G. B. (eds.). (2016). “Creativity in higher education: the use of creative cognition in studying,” in Psychology of Creativity: Cognitive, Emotional, and Social Processes (Hauppauge, NY: Nova Science Pub Inc.), 3–20. Available online at: https://www.novapublishers.com/catalog/product_inf...

[B50] Schuur W. A. Baumgartner S. E. Sumter S. R. Valkenburg P. M. (2015). The Consequences of media multitasking for youth: a review. Comput. Hum. Behav. 53, 204–215. 10.1016/j.chb.2015.06.035

[B51] Seddon A. L. Law A. S. Adams A. M. Simmons F. R. (2021). Individual differences in media multitasking ability: the importance of cognitive flexibility. Comput. Hum. Behav. Rep. 3:100068. 10.1016/j.chbr.2021.100068

[B52] Sternberg R. J. (2000). Images of mindfulness. J. Soc. Issues 56, 11–27. 10.1111/0022-4537.00149

[B53] Tursynkulova E. Madiyarov N. Turlybek S. Peruza D. (2023). The effect of problem-based learning on cognitive skills in solving geometric construction problems: a case study in Kazakhstan. Front. Educ. 8:1284305. 10.3389/feduc.2023.1284305

[B54] Tymofiyeva O. Gaschler R. (2020). Training-induced neural plasticity in youth: a systematic review of structural and functional MRI studies. Front. Hum. Neurosci. 12:497245. 10.3389/fnhum.2020.497245 33536885 PMC7848153

[B55] Uncapher M. Lin L. Rosen L. Kirkorian H. Baron N. Bailey K.. (2017). Media multitasking is associated with cognitive, psychological, neural, and learning differences. Pediatrics: Off. J. Am. Acad. Pediatrics 22, s62–s66. 10.1542/peds.2016-1758D 29093034 PMC5658797

[B56] Verghese A. Garner K. G. Mattingley J. B. Dux P. E. (2016). Prefrontal cortex structure predicts training-induced improvements in multitasking performance. J. Neurosci. 36, 2638–2645. 10.1523/JNEUROSCI.3410-15.2016 26937005 PMC6604864

